# Full-dose hepatitis B virus vaccination coverage and associated factors among health care workers in Ethiopia: A systematic review and meta-analysis

**DOI:** 10.1371/journal.pone.0241226

**Published:** 2020-10-27

**Authors:** Nefsu Awoke, Henok Mulgeta, Tsegaye Lolaso, Tiwabwork Tekalign, Serawit Samuel, Mohammed Suleiman Obsa, Robera Olana

**Affiliations:** 1 Department of Nursing, College of Health Science and Medicine, Wolaita Sodo University, Wolaita Sodo, Ethiopia; 2 Department of Nursing, College of Health Science, Debre Markos University, Debre Markos, Ethiopia; 3 School of Public Health, College of Health Science and Medicine, Wolaita Sodo University, Wolaita Sodo, Ethiopia; 4 Department of Anesthesia, College of Health Science and Medicine, Wolaita Sodo University, Wolaita Sodo, Ethiopia; Kaohsiung Medical University, TAIWAN

## Abstract

**Introduction:**

The hepatitis B vaccine is the backbone of hepatitis B prevention. All health care workers must receive a full-dose (3-dose vaccine series) to achieve >90% protection against hepatitis B virus. There is limited evidence available on vaccination coverage of HBV among health care workers in Ethiopia. Therefore, the objective of this study was to estimate the national full-dose hepatitis B vaccination coverage and the associated factors among health care workers in Ethiopia.

**Methods:**

Studies were retrieved from PubMed, EMBASE, Web of Science, SCOPUS, CINAHL, and Google Scholar by using a combination of search terms with Boolean operators. The quality of each study was evaluated independently by three authors using the modified Newcastle-Ottawa Scale (NOS) for cross-sectional studies. Statistical analyses were performed using STATA™ Version 14 software. Meta-analysis was carried out using a random-effects (DerSimonian and Laird) method. The heterogeneity test was conducted by using I-squared (*I*^*2*^*)* statistics. Leave-one-out sensitivity analysis was performed.

**Results:**

A total of 15 articles with 5734 participants were included in this systematic review and meta-analysis. The pooled prevalence of full-dose hepatitis B virus vaccination coverage among health care workers in Ethiopia was 20.04% (95% CI: 13.83, 26.26); *I*^*2*^ = 98.9%). Being male sex (*p* = 0.002), having work experience of less than 5 years (*p* < 0.001), educational level of diploma and below (*p* = 0.003), health care providers who received training on infection prevention (*p* < 0.001), and those who had a history of exposure to blood and body fluids (*p* = 0.001), were factors significantly associated with full-dose hepatitis B virus vaccination.

**Conclusion:**

The national full-dose hepatitis B vaccination coverage among health care workers was low. Training of health care workers in infection prevention, particularly in hepatitis B and testing and providing hepatitis B vaccination for newly recruited staff and every 5 years for those long-term workers were recommended to increase the uptake of the vaccine.

## Introduction

Hepatitis B is an infectious disease caused by a virus called the hepatitis B virus (HBV) and can cause both acute and chronic diseases [1]. Although there are different prevention strategies for hepatitis B, such as standard (universal) precautions and enhanced percutaneous injury precautions, such as double-gloving in surgery [2] the hepatitis B vaccine is the backbone of hepatitis B prevention [3]. The CDC recommends that all health care workers receive a 3-dose vaccine series with an approximate protection rate of 30%–55% after the first dose, 75% after the second dose, and >90% after the third dose in adults aged ≤40 years [4].

Hepatitis B virus infection is one of the serious challenging blood-borne occupational hazards among health care workers [5–9]. They are exposed to a higher risk of blood-borne pathogens and other body fluids, such as saliva, menstrual, vaginal, and seminal fluids, during their routine health care services [5, 6, 10]. The exposure might be a result of needle stick injuries, contamination with an infected patient's blood, a splashing of blood or other body fluids into the eyes, nose or mouth, or blood contact with non-intact skin [7, 10].

The estimated global prevalence of HBV in 2015 among the general population was 3.5%, with approximately 257 million persons living with HBV infection and 887 220 people’s dead in the same year [9]. In Africa, its prevalence was 6.1% in 2015, which was the highest across the globe [9]. Moreover, the prevalence of HBV ranged from 5% to 8% [11] in the Sub-Saharan region. Additionally, the estimated prevalence of hepatitis B virus (HBV) among the general population in Ethiopia was 6% [12].

HBV/HIV co-infection accounts approximately 5–20% of people infected with HIV even though it varies significantly between risk-based groups, regions and, reflecting different patterns of transmission [13]. In Sub Saharan Africa the prevalence of HIV/HBV co-infection was reported to be 15%; whereas in Ethiopia the documented prevalence of co infection hospital-based studies was 3. 9–14% [14].

Ethiopia launched an expanded program on immunization (EPI) with targeting children of under-one year of age and women of reproductive age group (15–49 years age) in 1980 comprising of with six antigens namely BCG, Diphtheria, pertussis, tetanus, polio and measles [15, 16]. In 2007 the government added Hepatitis B and Hib vaccines to the standard EPI schedule [16]. The immunization cost was largely covered by donors and the main funding partners were USAID, UNICEF and GAVI Alliance. Although, the government funds some traditional routine vaccine, such as TT, BCG, and 50% of Polio vaccine [16].

Compared to the general population the risk of HBV infection is fourfold greater among HCWs [9, 17]. Worldwide, there are approximately 35 million health workers [10]. Of these, approximately 5.9% of them were exposed to HBV every year [18]. The annual HBV infection rate is estimated to be 70 000, and more than 90% of these infections occur in developing countries [10]. The prevalence of HBV infection among health workers ranges from 0.8% to 74.4%, although it depends upon the region where they work [11]. In Ethiopia, the pooled national prevalence of HBV among health care workers is estimated to be 5% [12].

Different strategies and guidelines were implemented to reduce the risk of HBV infections among healthcare workers in Ethiopia; for instance, the Ethiopian Federal Ministry of Health (FMOH) infection-prevention guidelines recommend that all health care workers be vaccinated against HBV before they begin clinical attachments; Despite this the prevalence of hepatitis B vaccination remains low [19, 20].

Hepatitis B vaccination remains the most effective and feasible method to prevent HBV infection to and from HCWs and their patients [5, 6, 18]. The World Health Organization (WHO) recommended that all HCWs should be vaccinated against HBV [5]. However, approximately 24% of global health care workers remain unvaccinated [11]. According to the WHO report, only 18–39% of health care workers in low and middle–income countries (LMICs) received HBV vaccination as compared to 67–79% in high-income countries [21]. In the United States and China overall, 63.4% and 60% of HCW received complete ≥3 doses of hepatitis B vaccination respectively [18, 22]. In Africa, only a quarter of HCWs were fully vaccinated for HBV, with an estimated full hepatitis B vaccination coverage of 24.7% [6].

Although HBV vaccination is critical to prevent HBV infection among health care workers, limited evidence is available on vaccination coverage of HBV among health care workers in Ethiopia. Therefore, the objective of this study was to estimate the national full-dose hepatitis B vaccination (uptake of ≥3 doses of vaccine) coverage and the associated factors among health care workers in Ethiopia.

## Materials and methods

### Study design and search strategy

We systematically searched PubMed, EMBASE, Web of Science, SCOPUS, CINAHL, and Google Scholar for published articles. Unpublished studies were searched from the Addis Ababa University research repository. The core search terms and phrases were “Health care workers”, “Health care professionals”, “Hepatitis B vaccination”, “Hepatitis B vaccine”, “Vaccination status”, “Vaccination coverage”, “Hepatitis B antibody”, and “Ethiopia”

The following search terms with Boolean operators were used to search related articles: (((Health care workers OR Health Care Personnel OR health care providers OR health care professionals OR Nurse OR Medical doctors OR Midwifery OR Surgeon OR Midwife OR Physician OR Health office OR Lab technician OR General practitioner OR Pharmacist OR Specialist OR Dental doctor)) AND (Hepatitis B OR Hepatitis B status OR Hepatitis B vaccination coverage OR Hepatitis B vaccine OR uptake OR vaccination OR coverage OR immunization OR Hepatitis B virus OR Vaccines OR HBV OR Vaccination status OR Vaccination coverage OR Immune response OR vaccine efficacy OR Hepatitis B antibody)) AND Ethiopia

The report was written according to the Preferred Reporting Items for Systematic Reviews and Meta-Analyses (PRISMA) guidelines [23]. EndNote (version X8) reference management software for Windows was used to download, organize, review, and cite related articles.

### Study selection and eligibility criteria

#### Publication status

We included both published and unpublished studies.

#### Study area

Ethiopia.

#### Language

Studies written in the English language.

#### Study period

Studies conducted or published from 2007 to April 30, 2020.

#### Study type

All observational studies reported the prevalence of complete vaccination against the hepatitis B virus among health care workers.

#### Study content

Studies that reported the prevalence of full-dose vaccination against the hepatitis B virus among health care workers.

Studies were excluded if:

1. Studies that did not report the prevalence of health care workers who take a full-dose of hepatitis B vaccination
2. Methodologically poor studies

### Study extraction and quality appraisal

The data were extracted by three independent authors (N.A, H.M, and T.L) using a data extraction format prepared in a Microsoft Excel 2013 spreadsheet. The extracted data were: the first author’s name, publication year, region, design, sample size, sampling method, prevalence of full-dose vaccination against hepatitis B, and associated factors with their odds ratio. The quality of each study was assessed using the modified Newcastle-Ottawa Scale (NOS) for cross-sectional studies [24]. Studies were included with a score of 7 and more on the NOS. The quality of each study was evaluated independently by three authors (N.A, H.M, and T.L) and any disagreements that appeared during abstraction were resolved by discussion and consensus.

### Outcome variable and operational definition

The outcome of this review was full-dose hepatitis B virus vaccination. Full-dose hepatitis B virus vaccination was defined as healthcare workers who had received three HBV vaccine doses intramuscularly at 0, 1, and 6 months [4].

### Statistical analysis

Statistical analyses were performed using STATA™ Version 14 software. The heterogeneity test was conducted by using I-squared (*I*^*2*^*)* statistics. The value of *I*^*2*^ statistics ranges from 0 to 100% and 25, 50, and 75%, which represent low, medium, and high heterogeneity across the included studies, respectively [25]. The pooled prevalence of full-dose vaccination against the hepatitis B virus among health care workers was carried out using a random-effects (DerSimonian and Laird) method. To minimize the potential random variations between studies; the sources of heterogeneity were analyzed using subgroup analysis, and meta-regression. A leave-out-one sensitivity analysis was conducted by excluding each study at a time to identify the impact of each study on the overall estimate.

### Study selection

**Fig 1:**
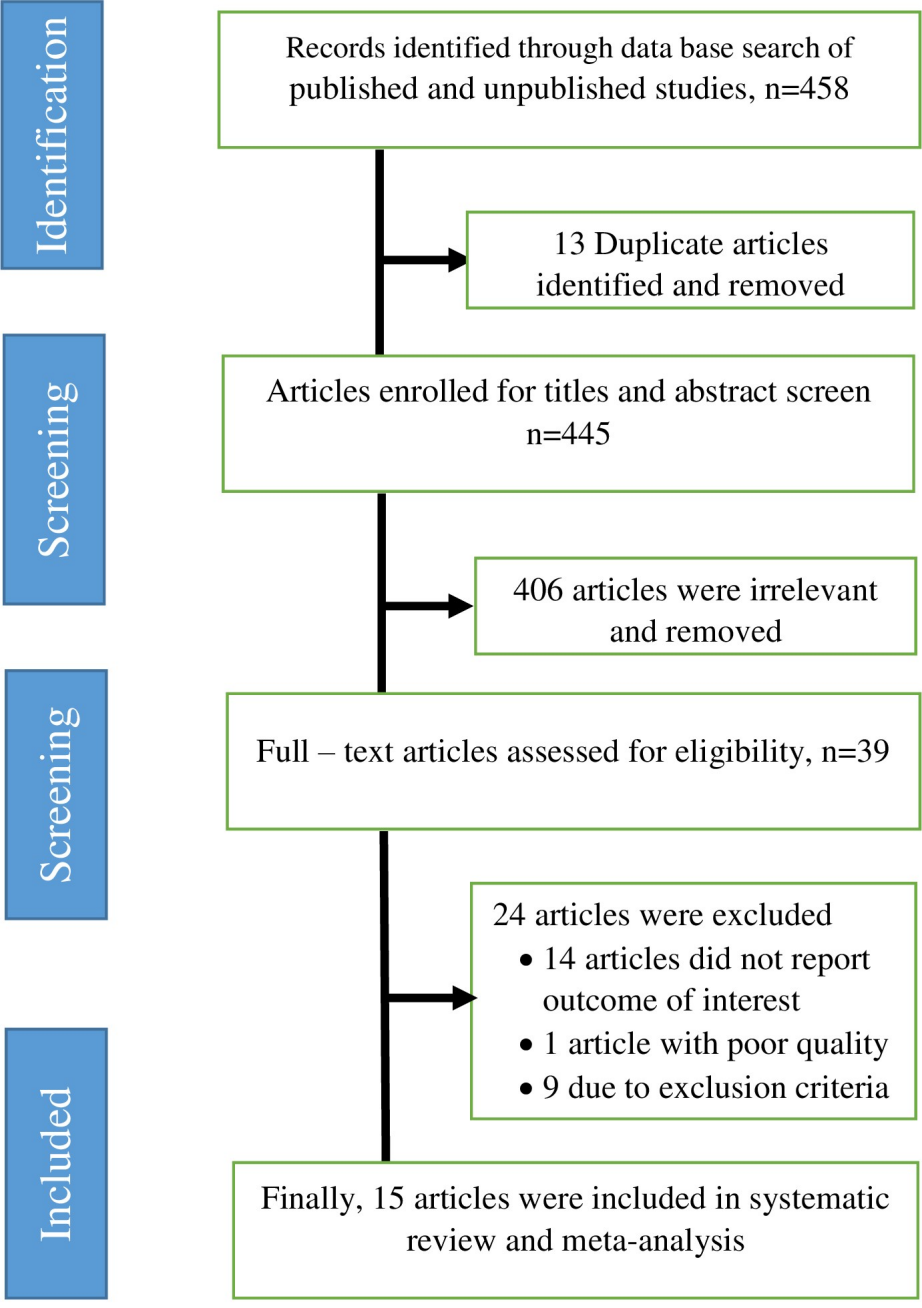
PRISMA flowchart diagram of the study selection.

Initially, 458 articles were retrieved through electronic online and manual searching. Of these, 13 duplicate articles were found and removed. Four hundred forty-five irrelevant articles were excluded by title and abstract screening. The remaining 39 full articles were assessed for eligibility; among them, 14 articles did not report the outcome of interest, 1 article with poor methodological quality and 9 articles that did not report the prevalence of health care providers who take a full-dose of hepatitis B vaccination were excluded. Finally, a total of 15 articles were included in the systematic review, and meta-analysis (Fig 1).

### Study characteristics

A total of 15 articles with 5734 participants were included in this systematic review and meta-analysis. All the included studies were cross-sectional studies and the sample size ranged from 232 [26] to 1125 [27]. Regarding the regional distribution of the included studies, 4 were conducted in the Oromia region [26, 28–30], 3 in the Amhara region [22, 27, 31], 5 in Addis Ababa [33–37], 2 in the Southern Nations, Nationalities and Peoples Region (SNNPR) [38, 39], and 1 study in Diredawa [40]. Among the included studies, the prevalence of full-dose hepatitis B vaccination coverage among health care workers ranged from 1.3 [26] to 62.7 [38] (Table 1)

**Table 1:** Characteristics of the included studies in the systematic review and meta-analysis for the prevalence of full-dose hepatitis B vaccination coverage among health care workers in Ethiopia, 2020.

No	Author/s name (Reference)	Publication year	Study area	Region	Study design	Sample size	NOS	Prevalence %(95%CI
1	Abebaw T. et al., [28]	2017	Shashemene	Oromia	Cross sectional	423	9	12.9 (9.71, 16.09)
2	Abeje G. and Azage M. [31]	2015	Bahirdar city	Amhara	Cross sectional	370	8	5.4 (3.09, 7.70)
3	Afework G. [33]	2015	Addis Ababa	Addis Ababa	Cross sectional	240	7	4.4 (1.81, 6.99)
4	Akibu M. et al. [29]	2018	Adama	Oromia	Cross sectional	386	9	25.6 (21.25, 29.95)
5	Bante A. et al. [38]	2018	W/Sodo	SNNPR	Cross sectional	370	8	62.7 (57.77, 67.63)
6	Bedaso A. et al., [39]	2018	Hawassa	SNNPR	Cross sectional	241	8	21.9 (16.68, 27.12)
7	Belete T. [34]	2016	Addis Ababa	Addis Ababa	Cross sectional	394	8	16.5 (12.83, 20.17)
8	Biset A. and Adugna H. [32]	2017	Gonder	Amhara	Cross sectional	286	9	28.7 (23.46, 33.94)
9	Dugassa M., [30]	2018	East Wollega	Oromia	Cross sectional	500	9	34.2 (30.04, 38.36)
10	Feleke B. [27]	2016	Amhara	Amhara	Cross sectional	1125	9	4.0 (2.86, 5.15)
11	Girma T. [26]	2015	West Hararghe	Oromia	Cross sectional	232	8	1.3 (0.16, 2.76)
12	Lateef I. [35]	2018	Addis Ababa	Addis Ababa	Cross sectional	300	9	40.3 (34.75, 45.85)
13	Mekonnen R. et al. [40]	2018	Dire dawa	Dire dawa	Cross sectional	282	8	36.2 (30.59, 41.81)
14	Taddesse G. et al., [36]	2016	Addis Ababa	Addis Ababa	Cross sectional	313	9	1.6 (0.21, 2.99)
15	Yimer S. et al. [37]	2017	Addis Ababa	Addis Ababa	Cross sectional	272	8	8.8 (5.43, 12.17)

### Prevalence of full-dose hepatitis B vaccination coverage among health care workers

**Fig 2:**
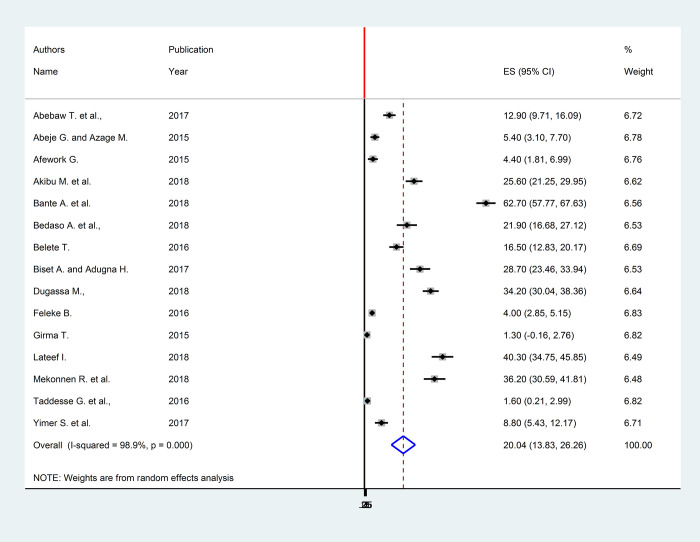
Forest plot showing the pooled prevalence of full-dose hepatitis B vaccination coverage among health care providers in Ethiopia.

A DerSimonian and Laird random-effects model was fitted to determine the pooled effect size. Accordingly, the national pooled prevalence of full-dose hepatitis B vaccination coverage among health care workers with a random-effects model was 20.04% (95% CI: 13.83, 26.26) with a heterogeneity index (*I*^*2*^) of 98.9% (p < 0.001) (Fig 2).

### Subgroup analysis

**Fig 3:**
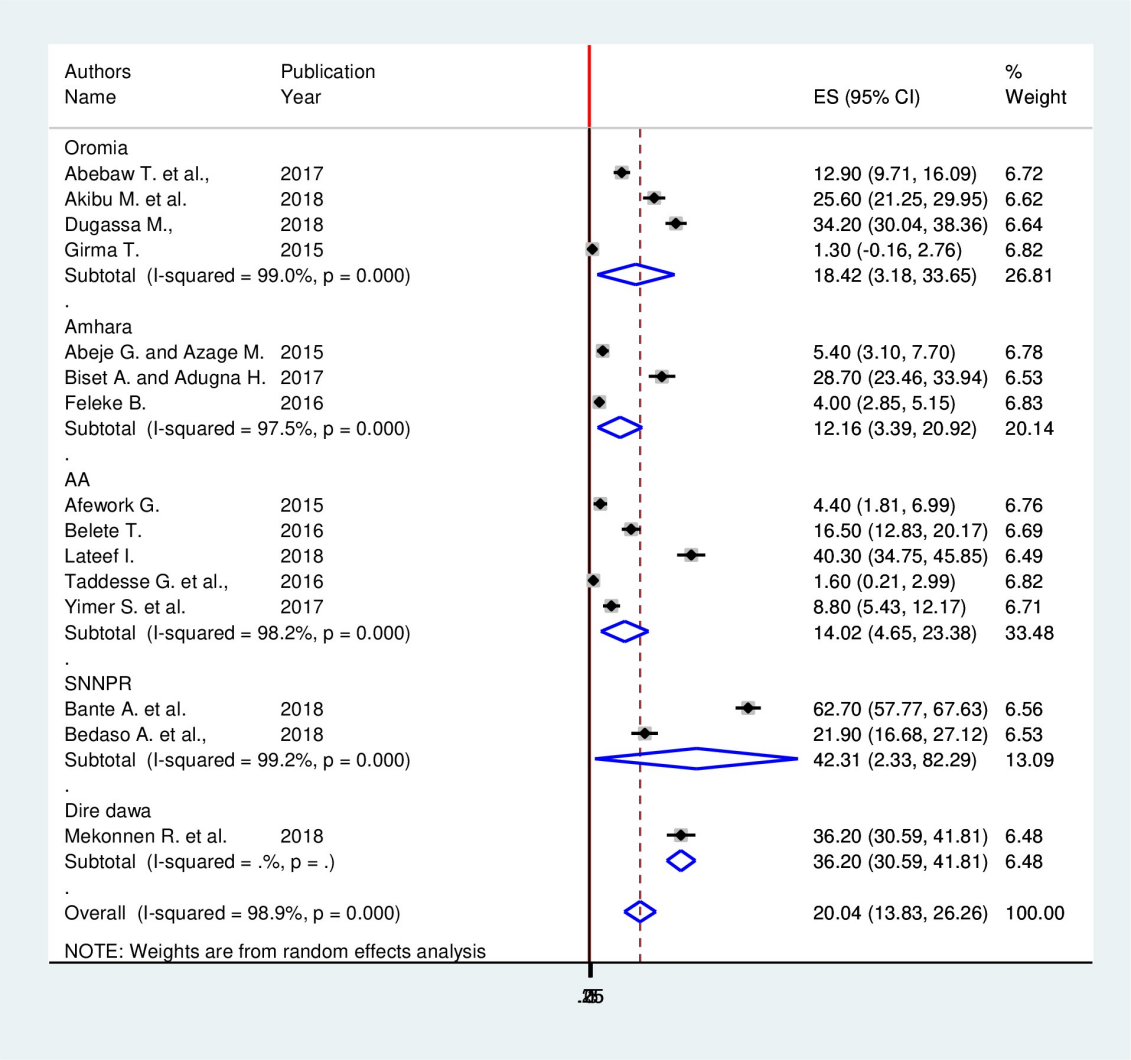
Subgroup analysis of full-dose hepatitis B vaccination coverage among health care providers by region in Ethiopia.

Since this meta-analysis showed a considerable heterogeneity, subgroup analysis was done using region where the studies were conducted. Based on this, the highest (42.33%; 95% CI: 2.33, 82.29), *I2* = 97.5%) and the lowest (12.16%; 95% CI: 3.39, 20.92), *I2* = 97.5%) prevalence of HBV full-dose vaccination among healthcare workers was observed in the SNNPR and Amhara region, respectively. In Oromia region pooled prevalence of full-dose hepatitis B vaccination coverage was 18.42% (95% CI: 3.18, 33.65) with a heterogeneity index (*I*^*2*^) of 99.9% (p < 0.001). In Addis Ababa the documented prevalence was 14.02% (95% CI: 4.65, 23.38) with a heterogeneity index (*I*^*2*^) of 98.2% (p < 0.001) (Fig 3).

### Heterogeneity and publication bias

**Fig 4:**
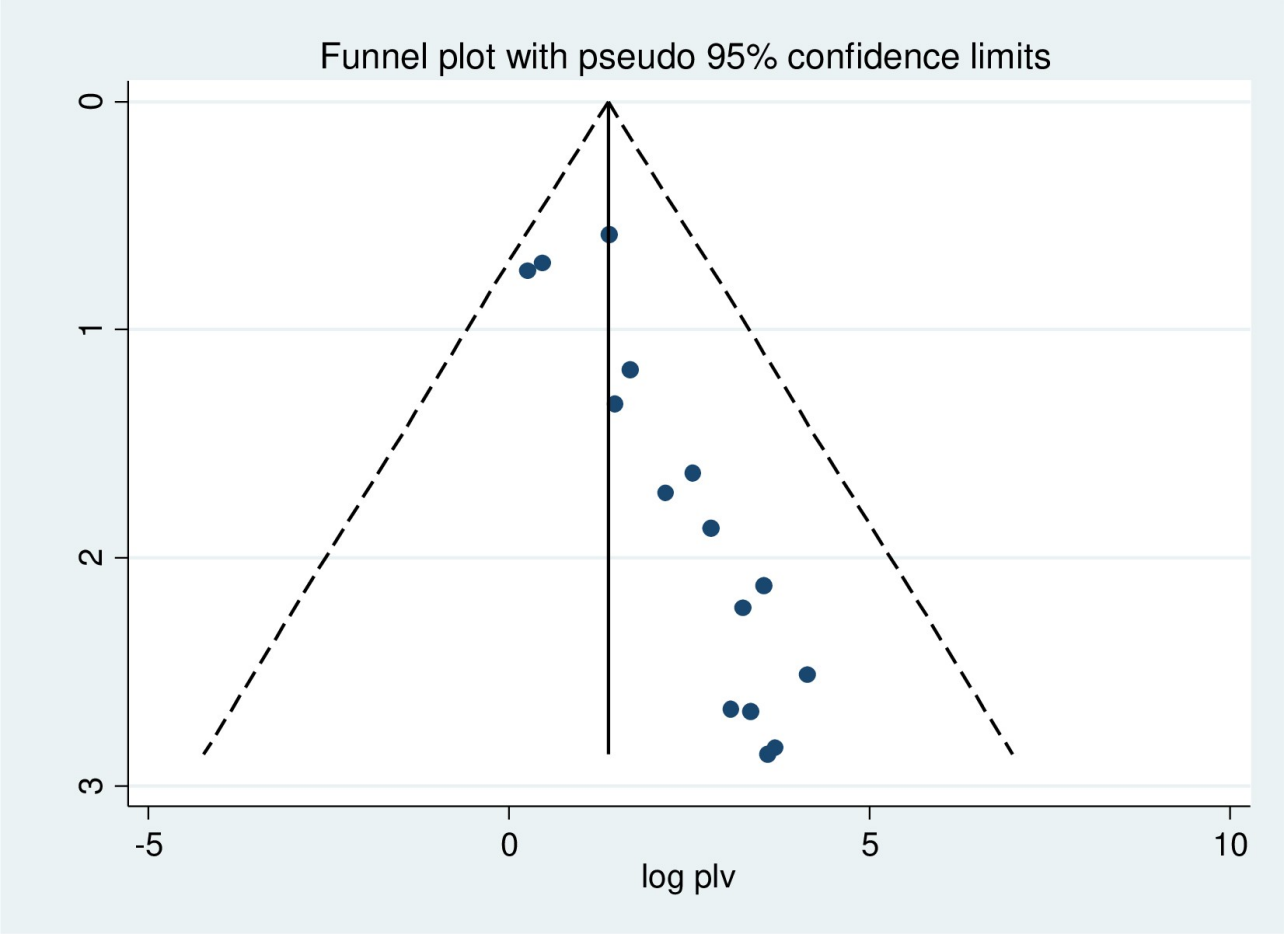
Funnel plot to test the publication bias in 15 studies with 95% confidence limits.

To adjust and minimize the reported heterogeneity of this study (*I*^*2*^ = 98.9%), we performed a subgroup analysis based on the region in Ethiopia. Meta-regression was also conducted to identify the source of heterogeneity using sample size and year of publication as a covariate. It was indicated that there was no effect of sample size and year of publication on heterogeneity between studies (Table 2). The presence of publication bias was tested by Egger’s test, and graphically by a funnel plot. Visual inspection of the funnel plot indicated asymmetrical distribution (Fig 4), which is statistically significant as evidenced by the Egger test (*p* < 0.001). Therefore, trim and fill analysis using the random-effects model was performed to determine the final effect size. However, a similar effect size was obtained using the model. Furthermore, we executed sensitivity analysis by removing studies step by step to evaluate the effect of a single study on the overall effect estimate. The results indicated that removing a single study did not have a significant influence on pooled prevalence (Table 3).

**Table 2:** Meta-regression analysis of factors affecting between-study heterogeneity.

Heterogeneity source	Coefficients	Std. Err.	P-value
Publication year	4.232	2.659	0.137
Sample size	-0.002	0.006	0.814

**Table 3:** Sensitivity analysis of pooled prevalence for each study being removed one at a time.

Study omitted	Publication year	Estimate [95% CI]
Abebaw T. et al.,	2017	20.57(14.00, 27.14)
Abeje G. and Azage M.	2015	21.13(14.39, 27.88)
Afework G.	2015	21.20(14.53, 27.87)
Akibu M. et al.	2018	19.64(13.50, 26.32)
Bante A. et al.	2018	16.92(11.83, 22.00)
Bedaso A. et al	2018	19.91(13.50, 26.32)
Belete T.	2016	20.30(13.81, 26.80)
Biset A. and Adugna H.	2017	19.43(13.11, 25.75)
Dugassa M.,	2018	19.01(12.91, 25.10)
Feleke B.	2016	21.28(13.83, 28.73)
Girma T.	2015	21.45(14.47, 28.44)
Lateef I.	2018	18.61(12.49, 24.74)
Mekonnen R. et al.	2018	18.91(12.70, 25.12)
Taddesse G. et al.,	2016	21.44(14.38, 28.49)
Yimer S. et al.	2017	20.87(14.29, 27.44)

### Factors associated with full-dose hepatitis B vaccination coverage among health care workers

Nine variables were extracted to identify factors associated with full-dose hepatitis B vaccination coverage among health care workers. Of these, 5 variables (sex, experience, educational level, training on infection prevention, and history of exposure to blood and body fluids) were found to be significantly associated with full-dose HBV vaccination (Table 4). Depending on the sociodemographic characteristics of the study participants, male health care workers were 35% less likely to take full-dose hepatitis B vaccination than females (OR: 0.65 (95% CI 0.50–0.85), *p* = 0.002, *I*^*2*^: 44.3%; the heterogeneity test (*p* = 0.084) showed significant evidence of variation across studies, and Egger’s test showed no evidence of publication bias (*p* = 0.507)). Additionally, health care workers with an educational level of diploma and below were 53% less likely to take full-dose hepatitis B vaccination than those with an educational level of degree and above (OR: 0.47 (95% CI 0.29–0.77), *p* = 0.003, *I*^*2*^: 66.8%; the heterogeneity test (*p* = 0.017) showed no significant variation across studies, and Egger’s test showed no evidence of publication bias (*p* = 0.363)).

**Table 4:** Factors associated with full-dose hepatitis B vaccination coverage among health care workers in Ethiopia.

Determinants (References)	Comparison	Number of studies	Sample size	OR (95% CI)	*P* value	*I*^*2*^ (%)	Heterogeneity test (P value)	Egger test (P value)
Sex [27–29, 32, 33, 35, 37, 38]	Male Vs. Female	8	3203	0.65 (0.50–0.85)	0.002	44.3	0.084	0.507
Age [32–35, 37]	<30 Vs. ≥ 30 years	5	1436	0.73 (0.48–1.11)	0.139	58.7	0.046	0.076
Experience [28, 29, 32, 33, 35, 38]	<5 Vs. ≥ 5 years	6	1786	0.34 (0.23–0.51)	< 0.001	63.6	0.017	0.090
Profession [27–29, 32, 33, 34]	Nurses Vs. Others	7	3036	1.12 (0.60–2.10)	0.725	89.5	< 0.001	0.991
Marital status [27, 28, 32–35, 37, 38]	Married Vs. Others	8	3230	1.49 (0.95–2.33)	0.085	80.4	< 0.001	0.365
Educational level [30, 32, 33, 35, 37]	Diploma and below Vs. Degree and above	5	1433	0.47 (0.29–0.77)	0.003	66.8	0.017	0.363
Knowledge on HBV vaccine [28, 35, 37, 38]	Good Vs. Poor	4	1352	1.26 (0.46–3.45)	0.652	93	< 0.001	0.218
Training on IP [27, 29, 30, 33, 35, 37]	Yes Vs. No	6	2638	2.77 (1.61–4.76)	< 0.001	89	< 0.001	0.207
History of exposure [27, 29, 30, 35, 37]	Yes Vs. No	5	2417	2.93 (1.55–5.52)	0.001	86.8	< 0.001	0.237

Health care providers with less than 5 years of work experience were 66% less likely to take full-dose hepatitis B vaccination than those with more than 5 years of work experience (OR: 0.34 (95% CI 0.23–0.51), *p* < 0.001, *I*^*2*^: 63.6%; %, the heterogeneity test (*p* = 0.017) showed no significant variation across studies, and Egger’s test showed no evidence of publication bias (*p* = 0.090)). Health care providers who received training on infection prevention were almost three times more likely to take full-dose hepatitis B vaccination than those who lacked training (OR: 2.77 (95% CI 1.61–4.76), *p* < 0.001, *I*^*2*^: 89%; the heterogeneity test (*p* <0.001) showed no significant variation across studies and Egger’s test showed no evidence of publication bias (*p* = 0.207)). Those who had a history of exposure to blood and body fluids were almost three times more likely to take full-dose hepatitis B vaccination than those who had not (OR: 2.93 (95% CI (1.55–5.52), *p* = 0.001, *I*^*2*^: 89%; the heterogeneity test (*p* <0.001) showed no significant variation across studies, and Egger’s test showed no evidence of publication bias (*p* = 0.237)) (Table 4).

## Discussion

A systematic review and meta-analysis were conducted to estimate the level of full-dose HBV vaccination coverage among healthcare workers and associated factors in Ethiopia. The estimated pooled national prevalence of full-dose hepatitis B vaccination coverage among health care workers in Ethiopia was 20.04% (95% CI 13.83, 26.26). This result is lower than a similar systematic review and meta-analysis conducted in China and America, where the prevalence of vaccination coverage was 60% and 63.4% respectively [18, 22]. This might be due to differences in socioeconomic status and standards of health care services. Our estimate is also lower than other African countries such as Kenya, Nigeria, and Libya [6], but higher than the national prevalence of 11.4% of Cameron [41]. This might be because the cost and unavailability of vaccination might be the reason for the low coverage [6, 17]. Moreover, the tendency to be vaccinated against hepatitis B is increased in workplaces offering a free hepatitis B vaccine for their health care workers [18].

Based on the subgroup analysis, the highest 42.33% and the lowest 12.16 prevalence of HBV full-dose vaccination among healthcare workers was observed in the SNNPR and Amhara region, respectively. In Oromia region pooled prevalence of full-dose hepatitis B vaccination coverage was 18.42% whereas in Addis Ababa the documented prevalence was 14.02%. Difference might be because of the difference in sample size and number of included studies in this meta-analysis. For instance, the highest sample size included in this study was from Amhara region and the lowest number of studies were included from SNNPR.

The results of this meta-analysis should that sex, educational level, work experience, training on infection prevention, and history of exposure to blood and body fluids were significantly associated with full-dose HBV vaccination.

Male health care workers were 35% less likely to take full-dose hepatitis B vaccination than females. This is consistent with study conducted at a tertiary care hospital in India which showed higher vaccination rate among female HCWs [42]. This might be because women have higher risk perception than men, and women are more concerned to their health.

Health care workers having an educational level of diploma and below were 53% less likely to receive full-dose vaccination against hepatitis B as compared to those having an educational level of degree and above. This is consistent with studies conducted in the United States [43] Western Greece [44] and Nigeria [45]. This might be because there is better awareness of the infectious disease and a higher acceptance of vaccination among highly educated health care workers [46].

Health care providers who received training on infection-prevention were almost three times more likely to complete the vaccine than those who had not received it. This is consistent with a study conducted in China [18] and Zambia [47]. This is because the training of health care workers increases their awareness of the importance of being vaccinated against HBV and provides them with relevant information to scale up vaccine uptake since they are a high-risk population for HBV infection [6].

Additionally, this review found that health care workers with work experience of less than 5 years were 66% times less likely to complete full-dose hepatitis vaccination compared to those having work experience of greater than or equal to 5 years. This was in an agreement with a systematic review and meta-analysis performed in Africa among health care workers [6] and a study conducted in Italy [48] and Nigeria [34]. This might be because having a long duration of work experience results in repeated episodes of exposure to blood and body fluids that might assist them looking for vaccination against hepatitis B [6]. Moreover, health care workers who had a history of exposure to blood and body fluids were almost three times more likely to complete vaccination against hepatitis B than those who had no history of exposure. This is consistent with a study conducted in Tanzania [49].

Although this systematic review and meta-analysis provide up-to-date evidence regarding the national full-dose HBV vaccination coverage, there are some limitations that need to be considered in future research. First, the protocol of this study was not registered. Second, this study lacks studies from some regions of Ethiopia (Afar, Benishangul Gumuz, Harari, Somalia, and Tigray), and other regions have limited number of study (Diredawa have only one and SNNPR have two studies), therefore it may be difficult to generalize the findings to the national level. Third, we identified significant heterogeneity across the included studies and the presence of publication bias; thus, the interpretation of the results must be taken cautiously. Lastly, the included studies were based on self-reports of their vaccination status which may introduce recall and social desirability biases.

## Conclusions

This study revealed that one in five health care workers received the full-dose of HBV vaccination, which implies that the national full-dose of hepatitis B vaccination coverage among health care workers was low. Sex, educational level, work experience, training on infection prevention, and history of exposure to blood and body fluids were found to be significantly associated with full-dose hepatitis B vaccination coverage. Therefore, training health care workers on infection prevention particularly on hepatitis B, is recommended to increase the uptake of the vaccine. Although, testing HBsAg/anti-HBs for newly recruited health care providers, providing free vaccination for those HBsAg/anti-HBs negative workers, and also testing and providing vaccine every 5 years for those long-term workers is expected form the government and hospital authorities to scale up the utilization.

## Supporting information

S1 Checklist PRISMA 2009 checklist. (DOC)

S1 Data (XLSX)

## List of abbreviations

CI: Confidence Interval
EPI: expanded programme on immunization
FMOH: Federal Ministry of Health
HBV: hepatitis B virus
NOS: Newcastle Ottawa Scale
OR: Odds Ratio
PRISMA: Preferred Reporting Items for Systematic Reviews and Meta-Analyses
SNNP: *Southern Nations*, *Nationalities*, *and Peoples*
